# The International Medical Congress

**Published:** 1876-09

**Authors:** 


### Editorial.

### The International Medical Congress.

The Medical Congress was, as we anticipated, a complete success. A large and thoroughly representative body of medical men met in Philadelphia, and the result of their labors will reflect credit upon all who participated in the deliberations of the Congress. The place of meeting was appropriately at Philadelphia, the home of early American medicine, as well as the birth-place of the Declaration.

While, as a matter of course, the Congress was largely made up of American physicians, our foreign brethren were well represented, delegates being present from England, Ireland, Scotland Prussia, Russia, Austria, Denmark, Belgium, Norway, Finland, Australia, Japan, Cuba, the Argentine Republic, Mexico and Canada.

The addresses before the Congress in the general session were all of an excellent character; the address of welcome from Prof. Gross was particularly fine and was eloquently and impressively delivered. This being the "year of jubilee" to Americans, the natural tendency of the authors of most of the public addresses to make them of a historical nature was excusable; we are sure however, that the foreign delegates found much in them of interest, and will rejoice that so young a nation has done so much for the science of medicine and surgery. The addresses of Drs. Gray and Bowditch may be, perhaps, excepted as being not so much of a national or historical character, the teaching of both, if carried out, will have a large influence for good in an international as well as a national sense.

The selection of Prof. Gross as President, was a fitting compliment to the man and to the city in which the Congress was held; he presided with courtesy and dignity.

The arrangements for the meetings were admirably made and well carried out, the programme for each days work, the list of delegates registered, and the outlines of papers presented on the special questions assigned for discussions in the sections, which were furnished to each member, added much to the convenience of delegates. Admirable facilities were also placed at the service of the Congress for the reception of mail matter, and a special room, with ink and paper, was set aside for correspondence and conversation. With a eye to the convenience of the members whose places of residence was at a distance from the University who could not therefore conveniently go to lunch between the time of adjourment of the general session and the meeting of the sections, a lunch was served at one o'clock each day, in the basement. Everything passed off without clash or confusion ; the committee of arrangement of the American Medical Association might do well to take lessons.

The interest in the sections was well sustained, but centered, of course, in the three principle ones, Medicine, Surgery and Obstetrics.

The social element, while not made too prominent, added much to the pleasure of the week. The reception on Monday evening at Judges' Hall, the hospitable entertainment given by Drs. Wilson and Thompson on Tuesday evening, by Dr. Strawbridge on Wednesday evening, and by Messrs. H. C. Lea and J. B. Lippincott on Thursday evening, with the grand dinner Friday evening will be long remembered as very pleasant incidents of a memorable week. We can not now dwell upon all the points which we would delight to mention, but would refer our readers to the very full report which we are able to lay before them. As we were present during most of the session, we were able to secure such a report as will give a fair idea of what was done; we are sure however, that all our readers will desire a copy of the transactions when printed.

A resolution passed on the fifth day of the session, forbidding the members to furnish their papers, either as a whole or in abstract, to Medical Journals, seemed to us ill advised, and certainly too late in the session to accomplish its object.

———:o:———

The large space given to our report of the Congress crowds out several notices and reviews. They will appear in our next number.

